# Post-ischemic inflammation regulates neural damage and protection

**DOI:** 10.3389/fncel.2014.00319

**Published:** 2014-10-14

**Authors:** Takashi Shichita, Minako Ito, Akihiko Yoshimura

**Affiliations:** ^1^Department of Microbiology and Immunology, School of Medicine, Keio UniversityTokyo, Japan; ^2^Precursory Research for Embryonic Science and Technology, Japan Science and Technology AgencyTokyo, Japan

**Keywords:** damage-associated molecular patterns (DAMPs), inflammation, cytokines, inflammasome, resolution of inflammation

## Abstract

Post-ischemic inflammation is important in ischemic stroke pathology. However, details of the inflammation process, its resolution after stroke and its effect on pathology and neural damage have not been clarified. Brain swelling, which is often fatal in ischemic stroke patients, occurs at an early stage of stroke due to endothelial cell injury and severe inflammation by infiltrated mononuclear cells including macrophages, neutrophils, and lymphocytes. At early stage of inflammation, macrophages are activated by molecules released from necrotic cells [danger-associated molecular patterns (DAMPs)], and inflammatory cytokines and mediators that increase ischemic brain damage by disruption of the blood–brain barrier are released. After post-ischemic inflammation, macrophages function as scavengers of necrotic cell and brain tissue debris. Such macrophages are also involved in tissue repair and neural cell regeneration by producing tropic factors. The mechanisms of inflammation resolution and conversion of inflammation to neuroprotection are largely unknown. In this review, we summarize information accumulated recently about DAMP-induced inflammation and the neuroprotective effects of inflammatory cells, and discuss next generation strategies to treat ischemic stroke.

## INTRODUCTION

Inflammation is implicated in almost all of central nervous system (CNS) diseases (Lo, 2010; Moskowitz et al., 2010; Iadecola and Anrather, 2011). Neurodegeneration, infection, trauma, and ischemia stimulate immune responses in the brain, although to varying degrees. The process in neuronal injury involves various intracellular mechanisms (abnormal metabolism and degeneration of protein, dysfunction of organelles, etc.), which cause the activation of microglia and the infiltration of circulating immune cells (Lo, 2010). Inflammation may not be always main process in the pathology of CNS diseases; nonetheless, the distinct characteristics of ischemic stroke are large amount of necrotic neuronal death and extreme infiltration of immune cells (Moskowitz et al., 2010; Iadecola and Anrather, 2011).

Severe inflammation causes cerebral swelling, which is often fatal in ischemic stroke patients. Broad necrotic lesion generates abundant inflammatory mediators and damage-associated molecular patterns (DAMPs), which enhance the chemotaxis of circulating immune cells and make them more efficient participants to promote inflammation (Moskowitz et al., 2010; Iadecola and Anrather, 2011). Cerebral inflammation exaggerates vascular dysfunction and induces further neuronal cell death (Dirnagl et al., 1999). Thus, post-ischemic inflammation is an essential process in the pathophysiology of ischemic stroke and is closely related to the prognosis after stroke (Dirnagl et al., 1999; Lo, 2010; Moskowitz et al., 2010; Iadecola and Anrather, 2011). In addition, inflammation is generally considered useful for the clearance of the large amount of debris caused by brain cell necrotic death (Moskowitz et al., 2010; Iadecola and Anrather, 2011). Inflammation, resolution of inflammation, and repair of neural damage are sequential pivotal events after stroke. To clarify the detailed mechanisms of each step of cerebral inflammation is indispensable to develop next generation therapies for ischemic stroke. The molecular basis of these steps is now being clarified by the recent accumulating evidences. We summarize these findings and discuss the principles of post-ischemic inflammation from beginning to end.

## INFLAMMATORY DAMPs

**FIGURE 1:**
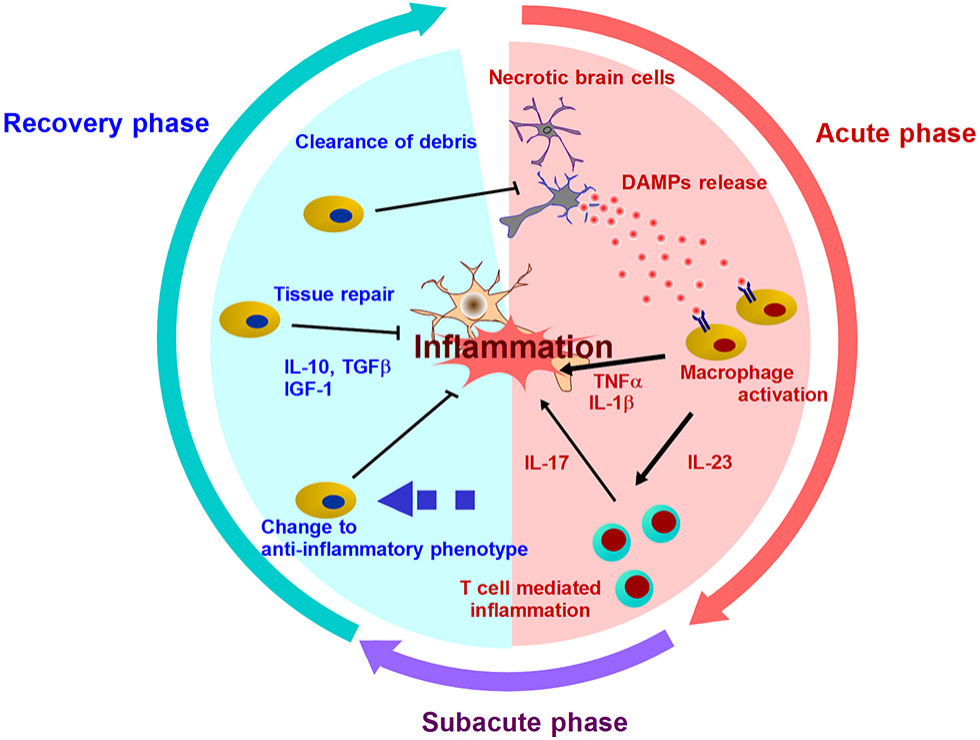
**Mechanisms of post-ischemic inflammation.** DAMPs are released into extracellular compartment and activate infiltrating immune cells by two ways: Signal 1 (via the activation of pattern recognition receptor) and Signal 2 (via the activation of inflammasome). Various inflammatory cytokines promote neuronal injury, and induce further inflammation mediated by T cells in subacute phase. After days and week after stroke onset, the resolution of post-ischemic inflammation is brought by the clearance of debris including DAMPs or inflammatory mediators, and the production of anti-inflammatory molecules or neurotrophic factors. In this recover phase, inflammatory immune cells turn into neuroprotective cells.

Brain ischemia induces various large metabolic changes in brain cells. Hypoxic stress, nutrients stress, and ER stress will cause cell death and trigger post-ischemic inflammation. Although receptors for pathogens such as Toll-like receptors (TLRs) are thought to be involved in early step of inflammation, brain is a sterile organ. Thus, endogenous molecules, i.e., DAMPs derived from injured brain cells, must trigger the inflammatory response in immune cells (**Table 1**). These DAMPs induce the activation of TLRs and other pattern recognition receptors [receptor for advanced glycation end products (RAGE) and c-type lectin receptors], which promote inflammatory mediator expression and tissue injury (**Figure 1**; Tang et al., 2007; Yanai et al., 2009; Suzuki et al., 2013). Recent scientific advances have suggested the existence of various types of DAMPs in ischemic brain.

**Table 1:** List of inflammatory DAMPs.

		DAMPs	Receptor	Reference
Signal 1	Nucleic acid	Mitochondrial DNA	TLR9	Zhang et al. (2010), Sun et al. (2013), Maeda and Fadeel (2014), Walko et al. (2014), Wenceslau et al. (2014)
		Self RNA, DNA	TLR7,9	Hyakkoku et al. (2010), Kawai and Akira (2010), Brea et al. (2011), Stevens et al. (2011), Leung et al. (2012)
	Lipid	Carboxyalkylpyrroles	TLR2	West et al. (2010)
		Oxidized phospholipids	CD36	Cho et al. (2005), Gao et al. (2006), Abe et al. (2010), Haider et al. (2011), Miller et al. (2011), Ho et al. (2012), Matt et al. (2013)
	Protein	HMGB1	TLR2,4, RAGE	Qiu et al. (2008), Zhang et al. (2011)
		Peroxiredoxin	TLR2,4	Shichita et al. (2012), Kuang et al. (2014)
		S100A8, A9	TLR4	Tsai et al. (2014)
		Mrp8, 14	TLR4	Loser et al. (2010)
		CIRP	TLR2,4	Qiang et al. (2013)
Signal 2	Nucleotide	ATP	P2X, P2Y	Martinon et al. (2002), Abulafia et al. (2009), Ceruti et al. (2009), Denes et al. (2013), Fann et al. (2013), Yang et al. (2014),
	Lipid	Phospholipids	?	Clemens et al. (1996), Bonventre et al. (1997), Muralikrishna Adibhatla and Hatcher (2006), Shanta et al. (2012), Iyer et al. (2013), Zhong et al. (2013)
	Protein	ASC specks	?	Baroja-Mazo et al. (2014), Franklin et al. (2014)

### NUCLEIC ACIDS AND NUCLEOTIDES

Various intracellular components are released into the extracellular space by necrotic brain cell death. Among these, nucleic acids and nucleotides are major DAMPs that have recently received much attention. Mitochondrial DNA released by cellular injury can be detected as DAMPs by immune cells, because mitochondria are considered to have a symbiotic origin that carries numerous characteristics resembling bacteria. Mitochondrial DNA is a sensor molecule of innate immunity by activating TLR9 and can be detected in cerebrospinal fluid after traumatic brain injury (Zhang et al., 2010; Walko et al., 2014). Vascular permeability is also increased by circulating mitochondrial DNA after injury (Sun et al., 2013; Wenceslau et al., 2014). Recently accumulated data indicates that mitochondrial DAMPs could be an important candidate for the trigger of post-ischemic inflammation, even if there is not yet any direct evidence (Maeda and Fadeel, 2014).

Self RNA and DNA (in complex with LL37 peptide) activate immune cells via TLR7 or TLR9 (Kawai and Akira, 2010). TLR7 is associated with the deterioration in ischemic stroke patients; in contrast, ischemic brain damage was not reduced in TLR9-deficient mice (Hyakkoku et al., 2010; Brea et al., 2011). Several reports demonstrate the implications of TLR7 and TLR9 in ischemic preconditioning. In these articles, the pretreatment using a TLR7 or TLR9 agonist reveals significant neuroprotection after cerebral ischemia by activating interferon regulatory factor 3/7- (IRF3/7)-induced type I interferon (IFN) signaling pathway (Stevens et al., 2011; Leung et al., 2012). Although the interaction between self nucleic acids and TLRs in the ischemic brain remains controversial, ischemic preconditioning via the TLR7 or TLR9 signaling pathway may represent a therapeutic strategy.

Purines (ATP and UTP) released from injured brain cells and their receptors, P2X and P2Y, function as alerting signals in CNS (Ceruti et al., 2009). Importantly, ATP also activates inflammasomes, which are large multimolecular complexes that control the activity of the proteolytic enzyme caspase-1 that cleaves pro-IL-1β to an active 17 kDa form (Martinon et al., 2002). The activation of the NLRP1 or NALP3 inflammasome has been recently reported to promote post-ischemic inflammation and neuronal death (Abulafia et al., 2009; Fann et al., 2013; Yang et al., 2014). Because IL-1β produced from both infiltrating immune cells and brain cells is important (Denes et al., 2013), it should be clarified how the inflammasome is activated in ischemic brain or hematopoietic cells. Inhibition of the inflammasome activation pathway may be a possible therapeutic strategy for ischemic stroke.

### LIPIDS

Various types of lipids are also important regulators of innate immunity. For example, oxidized low density lipoprotein (oxLDL) is a popular inflammatory mediator, which activates TLRs through binding with its receptor, CD36 (Stewart et al., 2010). Although the function of oxLDL in ischemic brain remains unclear, recent research has indicated that end products of lipid oxidation may be implicated in cerebral post-ischemic inflammation (Uchida, 2013). Carboxyalkylpyrroles, which are generated in inflammatory tissue, activate TLR2 and promote angiogenesis in ischemic organs (West et al., 2010). Oxidized phospholipids are also generated during cerebral inflammation and are considered to be DAMPs (Gao et al., 2006; Haider et al., 2011; Ho et al., 2012). Oxidized phospholipids are CD36 ligands that promote inflammation via TLR2 activation in ischemic brain (Cho et al., 2005; Abe et al., 2010). The recognition and endocytosis of oxidized lipids by pattern recognition receptors could regulate post-ischemic inflammation (Miller et al., 2011; Matt et al., 2013).

Phospholipids could also be inflammasome activators. Phospholipid metabolism is drastically altered by cerebral ischemia (Shanta et al., 2012). There are several reports showing the activation of phospholipase A2 (PLA2) in ischemic brain, which results in hydrolysis of membrane phospholipids (Clemens et al., 1996; Bonventre et al., 1997; Muralikrishna Adibhatla and Hatcher, 2006). Phospholipid hydrolysis and mitochondrial dysfunction induced by cerebral ischemia generate reactive oxygen species (ROS). Two recent studies have identified both ROS-dependent and ROS-independent pathways for inflammasome activation. The former is demonstrated by a charged phospholipid liposome that consecutively induces ROS-dependent calcium influx and NLRP3 inflammasome activation (Zhong et al., 2013). In the latter case, mitochondrial cardiolipin has been reported to directly bind to and activate the NLRP3 inflammasome (Iyer et al., 2013). Thus, the metabolism and modification of lipids during cerebral ischemia may be closely associated with the post-ischemic inflammation start signal.

### PROTEINS

High mobility group box 1 (HMGB1) and peroxiredoxin (Prx) family proteins are two major DAMPs in ischemic brain. There is a difference in the functional phase of these two proteins (Shichita et al., 2012). HMGB1, which is included in the nucleus of brain cells, is released extracellularly at the hyperacute phase (several hours after the stroke onset; Qiu et al., 2008). On the other hand, Prx family proteins function at the acute and subacute phases (12–72 h after the onset), especially in the penumbral area (Shichita et al., 2012). This is because Prx family protein expression is induced by an intracellular increase in ROS, which results from ischemic change. HMGB1 directly breaks down the blood–brain barrier and increases vascular permeability (Zhang et al., 2011). However, Prx directly induces the activation of infiltrating immune cells via TLR signaling. Ligustilide has been reported as a therapeutic candidate that suppresses cerebral post-ischemic inflammation by inhibiting the Prx/TLR4 signaling pathway (Kuang et al., 2014).

S100A8, S100A9, Mrp8, Mrp14, and cold-inducible RNA binding protein (CIRP) have also been reported to be protein DAMPs, although their relevance in post-ischemic inflammation has not yet been clarified (Loser et al., 2010; Qiang et al., 2013; Tsai et al., 2014). Inflammatory responses by these protein DAMPs occur through the activation of TLR2, TLR4, and RAGE. TLR2 and TLR4 signaling pathways are essential for sterile inflammation, including ischemic stroke (Chen et al., 2007). TLR2-blocking antibody is neuroprotective against ischemic brain injury (Ziegler et al., 2011). Similarly, resatorvid, which inhibits the TLR4 signaling pathway, attenuates ischemic brain injury and also suppresses Nox4-induced oxidative stress and neuronal apoptosis (Suzuki et al., 2012). It is also possible that DAMP-mediated TLR activation requires other adaptor molecules (Chun and Seong, 2010). CD14, a TLR4 co-receptor, may be implicated in post-ischemic inflammation (Reed-Geaghan et al., 2009). Heat shock protein gp96 is another candidate molecule that functions as an adaptor for both TLR2 and TLR4 (Yang et al., 2007).

It is not known whether protein DAMPs can activate inflammasomes. Recently, aggregated ASC (apoptosis-associated speck-like protein containing a caspase recruitment domain) has been reported to be released into the extracellular space after cell death and it activates inflammasomes in the surrounding immune cells (Baroja-Mazo et al., 2014; Franklin et al., 2014). Inflammasome activation, which occurs through ASC polymerization, results in caspase-1 activation and pyroptotic cell death. Extracellularly released ASC is internalized by surrounding macrophages and induces lysosomal damage and inflammasome activation. These mechanisms of inflammasome activation remain to be elucidated in ischemic brain injury.

### OTHER INFLAMMATORY DAMPs

Basic research may neglect the influence of aging and life habits by using healthy young rodents. These are important factors for the generation of DAMPs. Aging and continuous high serum glucose levels increase lipid peroxidation and AGEs in body systems (Basta et al., 2004; Cai et al., 2014). AGEs are proteins that are modified by sugar, through the Amadori and Maillard reaction. AGEs are found in chronic lesions; for example, the amyloid deposits that are surrounded by macrophages in patients with dialysis-related amyloidosis (Miyata et al., 1993). Thus, AGEs usually take a long time (more than a month) to generate; however, AGEs can be generated in a short period of time during inflammation (Weil, 2012). Glyoxal and glyceraldehyde induce AGE formation within 1 week (Takeuchi et al., 2001). In addition, the pivotal role of the RAGE in post-ischemic inflammation has been demonstrated (Muhammad et al., 2008). AGEs can be a potential DAMP, especially in aged human ischemic stroke patients.

## INFLAMMATION SUPPRESSION AND RESOLUTION

Activated immune cells and brain cells are the major players after various DAMPs trigger post-ischemic inflammation. These cells produce inflammatory cytokines, chemokines, and other cytotoxic mediators, and this leads to prolonged inflammation and progressive brain edema during several days after the stroke onset (**Figure 1**). However, post-ischemic inflammation rarely lasts for a long period of time, and the most intense inflammatory phase takes place within 7 days after stroke onset (Dirnagl et al., 1999; Iadecola and Anrather, 2011). In this phase, the number of infiltrating immune cells decreases remarkably, and remaining immune cells in ischemic brain produce anti-inflammatory or neurotrophic factors (Shichita et al., 2009; Smirkin et al., 2010). For example, the detailed mechanisms about the infiltration and the change to anti-inflammatory phenotype of neutrophils have been recently clarified (Cuartero et al., 2013; Gorina et al., 2014). The period of cerebral post-ischemic inflammation always ends, and thus, the mechanisms of its resolution must exist in ischemic brain.

Three major points on the resolution of inflammation have been discussed in a recent publication (Buckley et al., 2012). These points are the production of anti-inflammatory mediators, the depletion of inflammatory mediators, and the induction of anti-inflammatory immune cells. After post-ischemic inflammation, infiltrating macrophages turn into anti-inflammatory macrophages, which produce neurotropic factors and clear necrotic debris. Inflammatory DAMPs will also be implicated in the induction of anti-inflammatory macrophages, although its mechanism still remains to be clarified. We introduce recent advantages of the relationship between post-ischemic inflammation and its resolution.

### ANTI-INFLAMMATORY MEDIATOR PRODUCTION

Many molecules have been reported to be neuroprotective factors. However, most of these molecules failed to improve neurological deficits in ischemic stroke patients, even if they are effective in animal stroke models. It has been suggested that neuroprotection alone is not sufficient to improve the prognosis of human ischemic stroke. Anti-inflammatory mechanisms in the entire brain and how these mechanisms are triggered needs to be determined. Because most brain cells are dead in the ischemic region several days after the stroke onset, infiltrating immune cells and reactive glial cells could be major players in the tissue repair. In practice, accelerating their effect is a potential next generation therapeutic strategy, and direct *in vivo* reprogramming of reactive glial cells into functional neurons after cerebral injury by retroviral transduction of the NeuroD1 gene was recently reported (Guo et al., 2014).

IL-10 and TGF-β are major anti-inflammatory molecules in various organ injuries. Both are produced by infiltrating immune cells and reactive glial cells after ischemic brain injury. Viral overexpression of IL-10 in ischemic brain is neuroprotective (Ooboshi et al., 2006). One recent report demonstrated the anti-inflammatory effect of TGF-β by inhibiting excessive neuroinflammation during the subacute phase of brain ischemia (Cekanaviciute et al., 2014). Although the anti-inflammatory effects of IL-10 and TGF-β have been pivotal, it remains to be clarified whether these effects last up to 1 week after the stroke onset (Pál et al., 2012). If the mechanisms for stimulating TGF-β and IL-10 expression can be controlled, this may become a strong therapeutic method.

### DEPLETION OF INFLAMMATORY MEDIATORS AND CELLS

Infiltrating immune cells and reactive glial cells produce various inflammatory mediators. TNF-α and IL-1β directly induce neuronal cell death. IL-23 and IL-1β activate T cell-mediated innate immunity and promote secondary ischemic damage during the subacute phase of ischemic brain injury (Shichita et al., 2009; Konoeda et al., 2010). The existence of these inflammatory mediators, including DAMPs, prolongs post-ischemic inflammation and will be a threat to neuronal survival and repair. However, inflammatory mediator degradation mechanisms remain mostly unknown. Inflammatory molecules may be degraded by some enzymes or consumed by receptor-mediated endocytosis. It is expected that nucleotides and lipids are rapidly metabolized in the ischemic brain, and transfer by the blood stream or cerebrospinal fluid (CSF) will help to scavenge inflammatory mediator. Further research should clarify the detailed mechanisms to scavenge inflammatory molecules produced in the ischemic brain, and antibody therapy will be a pivotal therapeutic method targeting this potential mechanism. TNF-α and IL-23 neutralizing antibody have been used clinically for rheumatoid arthritis and psoriasis patients, respectively. Natalizumab is the neutralizing antibody for integrin-α4, which is necessary for T cell infiltration into the inflammatory tissue, and has already been used for multiple sclerosis (Yednock et al., 1992). T cell depletion from ischemic brain has received attention as a potential next generation therapy for ischemic stroke (Meisel and Meisel, 2011; Wei et al., 2011). Thus, antibody therapies may be used to help treat ischemic stroke patients in the near future.

Activation of inflammasomes in immune cells induces the production of IL-1β in its mature form, and finally results in the rapid cell death of the same cells, which is called pyroptosis. Pyroptosis may be a possible mechanism for the clearance of inflammatory immune cells. This is supported by the fact that dying cells detected using the TdT-mediated dUTP nick end labeling (TUNEL) staining method include macrophages and glial cells in the ischemic brain (Mabuchi et al., 2000).

## INDUCTION OF IMMUNE CELL REPAIR

The repair process for damaged brain tissues and regeneration of neural cells takes place during resolution of inflammation. It is difficult to separate this process from the anti-inflammatory mechanism, because they may overlap each other. We will further discuss neuroprotective factors and repairing the damage to immune cells.

### NEUROPROTECTIVE FACTORS

Various growth factors are also produced by immune cells and glial cells (Gudi et al., 2011). Among these, IGF-1 and FGF-2 are produced by infiltrating macrophages and microglia during the recovery phase of ischemic brain injury (which occurs 1 week after the stroke onset). IGF-1 and FGF-2 improve the neurological outcome by saving neuron and glial cells from cell death (Ness et al., 2004; Ikeda et al., 2005; Zhu et al., 2008; Hill et al., 2012; Lalancette-Hébert et al., 2012). IGF-1 also enhances repair after ischemic stroke by promoting neural regeneration, remyelination, and synaptogenesis (Lecker et al., 2007; Zhu et al., 2008, 2009; Kooijman et al., 2009). Further investigation should clarify the mechanisms of IGF-1 and FGF-2 induction in the ischemic brain. Recently, transfer of mesenchymal stem cells (MSCs) has been explored as a next generation therapy for ischemic stroke (Kalladka and Muir, 2014). MSCs produce various growth factors and promote neuronal survival and neurogenesis (Calió et al., 2014). By improving the transfer method, cell therapy may become a pivotal therapeutic strategy (Guo et al., 2013).

The neuroprotective effect of prostaglandin E2 (PGE2) and its receptor signaling pathway has received recent attention. PGE2 has an effect via four distinct G protein-coupled EP receptors (E-prostanoid: EP1, EP2, EP3, and EP4). The activation of EP2 signaling has a neuroprotective effect in ischemic brain injury, which was shown in the significant increase in infarct volume in mice lacking the EP2 receptor (McCullough et al., 2004). Similarly, signaling via the EP4 receptor, which is expressed in both neurons and ischemic endothelial cells, has neuroprotective effects against ischemic brain injury (Liang et al., 2011). The administration of an EP4 agonist reduces infarct volume and neurological deficits. In a neonatal hypoxic-ischemic encephalopathy model, the inhibition of EP1 receptor signaling or the activation of EP2, EP3, and EP4 receptor signaling reveals attenuation of the ischemic injury (Taniguchi et al., 2011). PGE2 and EP receptor signaling pathways have various functions, which are dependent on distinct pathology of cerebral diseases (Furuyashiki and Narumiya, 2011). Targeting specific EP receptors in ischemic brain may become a novel therapeutic method.

Similar to PGE2, some lipids have been reported to have anti-inflammatory effects and promote neuroregeneration (Serhan, 2014). Cerebral ischemia increases PLA2 activity, which results in the hydrolysis of phospholipids in the cellular membrane (Shanta et al., 2012). Although the PLA2 effect itself is cytotoxic because it disrupts the cellular membrane, PLA2 also generates docosahexaenoic acid (DHA) derivatives and lysophospholipids through phospholipid hydrolysis (Bonventre et al., 1997). Resolvin and Neuroprotectin have been investigated for their anti-inflammatory function in ischemic stroke (Marcheselli et al., 2003; Bazan, 2009). Lysophospholipids also increase in ischemic brain and promote neurite outgrowth (Ikeno et al., 2005; Spohr et al., 2011; Shanta et al., 2012). Regulating the effect of these lipids is expected for the resolution of post-ischemic inflammation.

### NEUROPROTECTIVE CELLS

Inflammatory DAMPs activate glial cells and infiltrating immune cells to promote post-ischemic inflammation. Paradoxically, this mechanism results in the infiltrating macrophage cell death and also induces anti-inflammatory and tissue-repairing immune cells.

Immune cell activation also induces anti-inflammatory cells. These cells have been called M2 macrophages, in contrast to the inflammatory M1 macrophages. Many researches have described the M2 macrophage markers; these markers include: arginase-1 (Arg1), chitinase3l3 (Ym), and Relmα (Fizz1). These markers are intracellular enzymes that are implicated in collagen synthesis and cell division; therefore, M2 enzymes are considered to promote tissue repair. Arg1 is the only marker that was reported to function as a neuroprotective enzyme (Estévez et al., 2006). However, these M2 markers may not be a good indicator for recovery after ischemic stroke. M2 markers are rapidly expressed in macrophages by TLR activation or other pattern recognition receptors, which also induce inflammatory cytokine expression (Hu et al., 2012). M2 markers appear in ischemic brain mostly during the same phase as the inflammatory mediators, the M1 markers, are expressed. In addition, the transfer of M2 marker positive-macrophages has not been reported to be sufficiently neuroprotective (Desestret et al., 2013).

During post-ischemic inflammation, some populations of macrophages and microglia become neuroprotective (Lalancette-Hébert et al., 2007). Galectin-1 has been suggested to be an inducer of anti-inflammatory macrophage/microglial cells (Starossom et al., 2012; Quintá et al., 2014). Galectin-1 is produced by astrocytes and has a neuroprotective effect against ischemic brain damage (Qu et al., 2011). Thus, the resolution of post-ischemic inflammation can be enhanced by the induction of a specific macrophage/microglial cell population. However, it is not clear whether the M2 markers truly reflect the neuroprotective function of macrophages and microglial cells. Suppressing inflammation alone is not enough to protect the brain from ischemic injury. IGF-1 and FGF-2 production seems to be a good index of repairing function (Lalancette-Hébert et al., 2012).

Further study is required to clarify whether sufficient clearance of inflammatory mediators (including DAMPs) begins neuronal regeneration after ischemic stroke. A recent study has suggested that there is a relationship between TLR activation and neuronal repair (Bohacek et al., 2012). It is possible that DAMPs triggers the secondary signals, which lead to resolution of post-ischemic inflammation, even if the primary signals via pattern recognition receptors promote ischemic damage. What is this mechanism? The role of immune cells, other than macrophages and microglia in part of the repair process, is not fully understood. This understanding may be critical for the establishment of next generation therapies for ischemic stroke.

## CONCLUSION

Immunity and various physiological mechanisms are implicated in the triggering, persistence, and resolution of post-ischemic inflammation. Recent accumulating evidences clarify the complexity of these mechanisms to understand the entire mechanisms. They will show promising potential targets to develop therapies for ischemic stroke.

## Conflict of Interest Statement

The authors declare that the research was conducted in the absence of any commercial or financial relationships that could be construed as a potential conflict of interest.
